# Predicting the occurrence of *Salmonella*, *Escherichia coli*, and pathogenic *E. coli* in agricultural ponds in the Georgia Coastal Plain

**DOI:** 10.1002/jeq2.70317

**Published:** 2026-11-02

**Authors:** J. Andrew Widmer, Matthew Stocker, Jaclyn E. Smith, Oliva Pisani, Alisa Coffin, Manan Sharma, Yakov Pachepsky, Laurel L. Dunn

**Affiliations:** ^1^ Department of Food Science and Technology University of Georgia Athens Georgia USA; ^2^ USDA‐ARS, Environmental Microbial and Food Safety Laboratory Beltsville Maryland USA; ^3^ USDA‐ARS, Southeast Watershed Research Laboratory Tifton Georgia USA

## Abstract

*Escherichia coli* is one measure of fecal contamination in water, which is expected to correlate with the presence of fecal pathogens, such as pathogenic *E. coli* or *Salmonella*. Supplementing *E. coli* measurements with other water quality measurements, for example, temperature or pH, may better identify situations when foodborne pathogens are likely to be present in water. This work used generalized and multinomial regression and machine learning (K‐nearest neighbors, random forest (RF), and support vector machines) to create explanatory models for levels of *E. coli*, pathogenic *E. coli*, and *Salmonella* presence in irrigation ponds. Model performance was mixed, with RF models performing best for *E. coli* levels (root mean square error [RMSE] = 0.45) for all ponds (*n* = 1266), pond 1 (RMSE = 0.44, *n* = 563), and pond 2 (RMSE = 0.71, *n* = 505), while generalized regression performed best at pond 3 (RMSE = 0.41, *n* = 198). Pathogenic *E. coli* presence for all ponds was best explained by RF (receiver operating characteristic area under the curve [ROC AUC] = 0.94, *n* = 717). *Salmonella* presence for ponds 1 and 2 (*n* = 284) was best explained by multinomial regression (ROC AUC = 0.83). These results indicate that explanatory models struggled to identify situations conducive to pathogen occurrence in this system and were not useful in identifying factors associated with pathogen presence.

## Plain Language Summary

Irrigation water is one of the inputs into fruit and vegetable production that is likely to introduce foodborne pathogens, but it is difficult to directly determine the presence of these pathogens. Indicators like *Escherichia coli* and turbidity are instead used to estimate if water is of suitable quality for use in produce operations. This work used machine learning to evaluate datasets collected over 27 months to determine what environmental and biotic factors affect the level of *E. coli* and presence of *Salmonella* and pathogenic *E. coli* in water samples. Some models performed well; random forests predicted *E. coli* level well for most datasets and performed best for pathogenic *E. coli*, while multinomial regression best predicted *Salmonella* presence. Model performance for the pathogen datasets was poor, with several missed positives, creating a dangerous situation where a pathogen is not expected, but is present.

**Abbreviations**
ARS: Agricultural Research Service
AUC: area under the curve
BAM: Bacteriological Analytical Manual
DO: dissolved oxygen
FDA: U.S. Food and Drug Administration
GHI: global horizontal irradiance
GLM: generalized linear model
KNN: K‐nearest neighbors
MAE: mean absolute error
MMSs: modified Moore's swabs
MNR: multinomial regression
MPN: most probable number
O157 CHROM: CHROMagar O157
PR: precision recall
RF: random forest
RFU: relative fluorescence units
RMSE: root mean square error
ROC: receiver operating characteristic
ROC AUC: receiver operating characteristic area under the curve
SC: *Salmonella* chromogenic
SMOTE: synthetic minority oversampling
SPC: specific conductance
STEC
: Shiga toxin‐producing *Escherichia coli*
SVM: support vector machines
TSB: tryptic soy broth

## 1 INTRODUCTION

### 1.1 Indicators of agricultural water suitability

Fresh produce remains an important vector of foodborne illness in the United States: 66.8% of *Escherichia coli* O157 (a Shiga toxin‐producing *Escherichia coli* [STEC]) illnesses and 42.6% of *Salmonella* illnesses are associated with produce (Interagency Food Safety Analytics Collaboration, 2024). Furthermore, irrigation water has been identified as a frequent route of introduction for these pathogens onto fresh produce (Gurtler & Gibson, 2022; Leaman et al., 2024), and pathogens such as Salmonella may persist beyond their initial introduction into a water source and are potentially introduced through irrigation throughout the season (H. Liu et al., 2018).

*E. coli* has long been used as an indicator of fecal contamination in water (Jang et al., 2017), particularly for water used in recreation areas (EPA, 2021) but was later briefly adopted by the U.S. Food and Drug Administration (FDA) for use in determining suitability for water used for irrigation of fresh produce (FDA, 2017, 2021b). *E. coli* alone as an indicator of foodborne pathogen presence is unreliable (Havelaar et al., 2017; Luo et al., 2015; Pachepsky et al., 2016), resulting in a 2024 FDA decision to no longer use generic *E. coli* level alone as a determinant of water quality. A grower‐conducted agricultural water assessment, based partially on qualitative and environmental factors to predict the risk of contamination to a water source, was introduced instead (FDA, 2024a). However, this new system may be subjective and provides growers with limited guidance to assess the safety of a water supply. Therefore, identifying more effective indicators to predict pathogen presence is a priority. Physical and chemical measures of water quality, from pH and turbidity to concentrations of specific metals, and biotic measures such as chlorophyll or phycocyanin may already be part of water monitoring and inform the kind of microbial life present in a water source (Swistock, 2022), and could be leveraged for assessing produce safety risk.

### 1.2 Requirements for agricultural water used for produce

Regulatory requirements for irrigation water used for fresh produce have shifted from decisions based on the amount of *E. coli*, a fecal indicator bacterium (FDA, 2022a), to a more wholistic analysis, which may be based on other biotic (e.g., crop characteristics) or abiotic factors (e.g., sunlight exposure) (FDA, 2021a, 2022b, 2024b). This could include modeling factors associated with increased pathogen prevalence. Models, both regression (generalized linear models [GLMs] and multinomial regression [MR]) and machine‐learning based (random forest [RF], support vector machines [SVM], and K‐nearest neighbors [KNN]), may provide a way to augment existing measures of water quality (Weller et al., 2021a) or reveal factors relevant to foodborne pathogen occurrence in water. This work seeks to develop models to explain the levels of *E. coli* across irrigation ponds and the occurrence of two important foodborne pathogens with the aim of providing better guidance on measuring and interpreting agricultural water quality.

### 1.3 Model selection

The models used in this study were selected based on performance in previous studies modeling pathogen or fecal indicator occurrence in irrigation water. Conditional RFs, KNN, penalized regression models, and SVM models were found to be effective predictors of both STEC and *Salmonella* in New York irrigation waters (Weller, Belias, et al., 2020). SVM and KNN have also been shown to have utility in predicting the occurrence of *Salmonella* in irrigation water samples (Polat et al., 2020). RF models and SVM have were effective in predicting the levels of *E. coli* in a spatial design like what was used in this study (Stocker et al., 2022). Models were selected to include a mix of traditional models and easy‐to‐explain machine learning models, so that results could be readily communicated to and applied by fresh produce growers.

## 2 MATERIALS AND METHODS

### 2.1 Study area and spatial collection of samples

Spatial design was based on previous work in the mid‐Atlantic (Pachepsky et al., 2018). Briefly, a sampling grid was overlaid on three different ponds (Figure 1A–C) in the Georgia Coastal Plain, including points along the perimeter and interior of the water body. Points were resampled approximately once a month from October through March, and twice times a month from April through September; collection took place from July 2021 through October 2023. Pond 1 had ca. 50 head of beef cattle rotated into the paddock immediately surrounding the pond. Pond 2 had no cattle access and was supplemented with ground water. More detailed description of the dynamics of Pond 2 is described by Albright et al. (2025). Pond 3 was located approximately 500 m north of Pond 2 on the same farm and fed by a separate creek; no ground water supplementation occurred in Pond 2.

**FIGURE 1:**
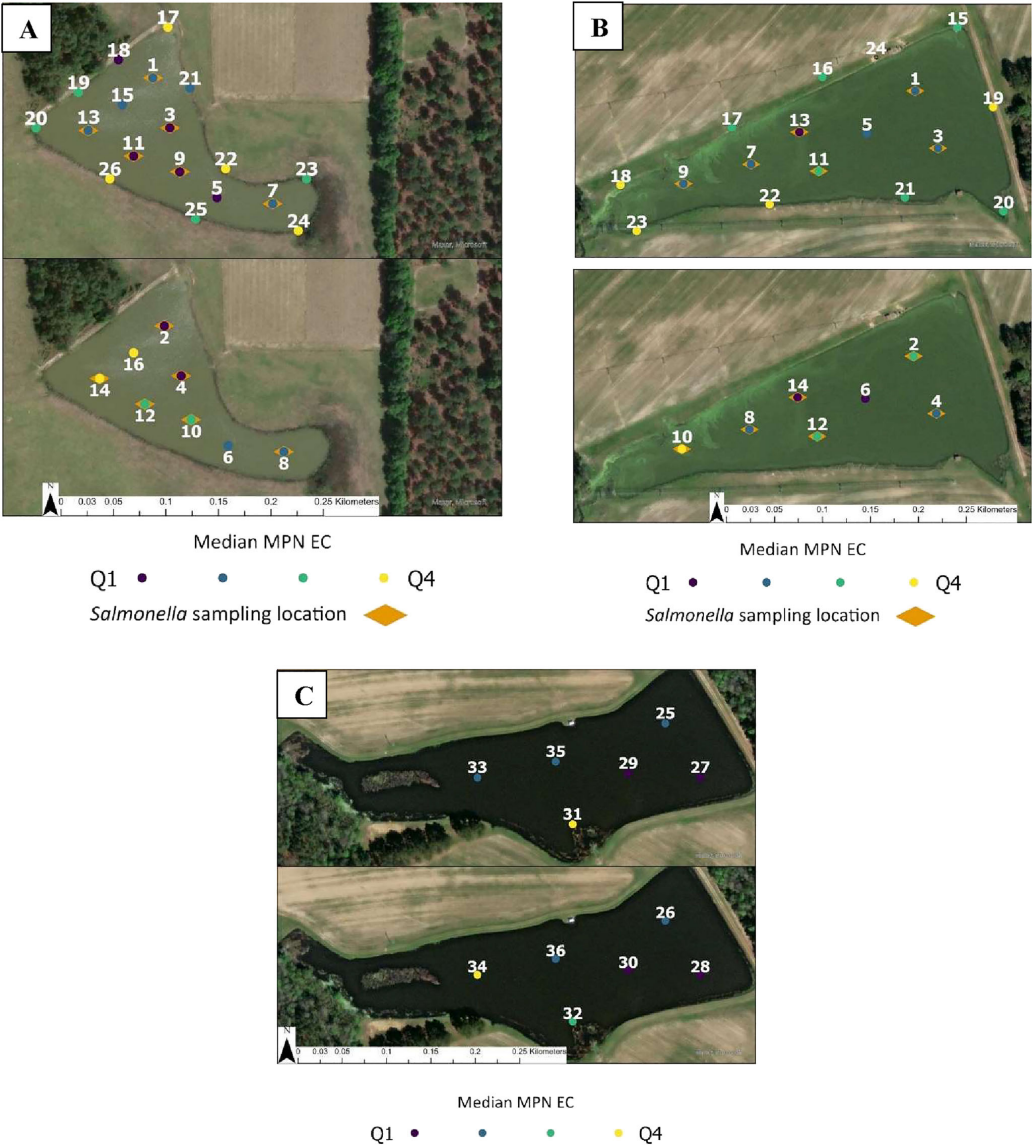
Sampling locations across the three ponds used in this study. (A) Pond 1, (B) Pond 2, and (C) Pond 3. The top image of each pond shows perimeter and interior surface sampling locations and the bottom interior subsurface. Points are colored according to the quartile of the median most probable number (MPN) *E. coli* (MPN *E. coli*) 100 mL^−1^. Orange diamonds behind the point indicate that the point was also sampled for *Salmonella*. No *Salmonella* samples were collected at Pond 3. *Source*: Imagery 2024 Maxar, Microsoft.

Global horizontal irradiance (GHI) (W/m^2^) values were retrieved for each site through the National Solar Radiation Database (Sengupta et al., 2018). Data for each farm were retrieved by selecting a 2 km × 2 km square including the farm's address. Weather data were taken from USDA Agricultural Research Service (ARS) weather stations located on each farm or nearby University of Georgia weather network stations when the ARS weather stations were inoperable.

### 2.2 Collection, enumeration, and isolation of *E. coli*

Water samples were collected and enumerated for *E. coli* as previously described (Widmer et al., 2025). For each sample, most probable number (MPN) *E. coli*100 mL^‐1^ was determined using Colilert reagent and Quanti‐tray 2000 MPN trays (IDEXX). Physicochemical water quality parameters were measured using an Exo2 multiparameter sonde (YSI) and included temperature (°C), pH, turbidity (NTU), phycocyanin levels (relative fluorescence units [RFU]; YSI Staff, 2019), chlorophyll‐a levels (RFU), fluorescent dissolved organic matter (RFU), dissolved oxygen (DO) (mg L^−1^), specific conductance (SPC) (µS cm^−1^), and nitrogen as nitrate (mg L^−1^).

After enumeration, up to five fluorescent (*E. coli*‐positive) and five yellow, nonfluorescent wells (*E. coli*‐negative, coliform positive) were selected. The foil side of each tray was sprayed with 70% ethanol and allowed to air dry. Wells was pierced with a sterile needle, and a 10 µm loopful streaked onto MacConkey agar (BD; fluorescent wells) or CHROMagar O157 (O157 CHROM; CHROMagar Microbiology; nonfluorescent wells). MacConkey plates were incubated overnight at 44.5°C, and O157 CHROM plates were incubated at 37°C for 24 h. A single typical *E. coli* or *E. coli* O157 colony was selected from each plate and grown overnight in tryptic soy broth (TSB; Difco) at 44.5°C.

### 2.3 Collection and isolation of *Salmonella*

Modified Moore's swabs (MMSs) were constructed as described by Sbodio et al. (2013) Briefly, a rolled segment of autoclaved cheesecloth was placed inside a section of PVC pipe with barb fittings at either end to allow for attachment of the hose from a peristaltic pump to force a known quantity of water through. At six interior sampling locations at Ponds 1 and 2 (indicated with orange diamonds in Figure 1A,B), 1 L was filtered through a MMS in addition to typical sampling, followed by a 1 m subsurface MMS sample collected at the same location, if water depth allowed for collection without disturbing sediment. MMSs were returned to their sterile roll‐top bags (Whirl‐Pak) and transported to the lab on ice in a cooler.

Upon receipt at the lab, MMSs were processed for *Salmonella* in a method adapted from Sharma et al. (2020). Briefly, the swab was removed from the PVC cassette and placed into 100 mL Universal Pre‐Enrichment Broth (Hardy Diagnostics) in a roll‐top bag and hand massaged for 1 min. Swabs were incubated for approximately 24 ± 2 h at 35°C before being processed according to the FDA's Bacteriological Analytical Manual (BAM) (Andrews et al., 2022). Briefly, 1 mL was transferred to 10 mL of tetrathionate (Neogen) broth and incubated overnight at 35°C, and 0.1 mL was added to 10 mL of Rappaport‐Vassiliadis (BD) broth and incubated 24 h at 42°C. After incubation, each broth was then streaked onto XLT‐4 agar (BD) and *Salmonella* chromogenic (SC) agar (Neogen). XLT‐4 plates were incubated at 35°C and checked for growth characteristic of *Salmonella* at 24 and 48 h. SC plates were incubated at 37°C and checked for growth at 24 h. Presumptive *Salmonella* growth was re‐streaked onto SC agar and incubated for 24 h at 37°C. Colonies were picked with a sterile loop, added to TSB, and incubated overnight at 37°C.

### 2.4 Preservation of bacterial isolates

A 0.6 mL aliquot of each TSB culture was mixed with 0.6  mL of 50% (v/v) glycerol /deionized water and stored at −80°C. Prior to analysis, samples were streaked onto CHROMagar *E. coli* (CHROMagar), O157 CHROM, or SC depending on the isolate being analyzed. *E. coli* K12, *E. coli* ATCC 35150, and *Salmonella enterica* Newport were used as control strains, respectively.

### 2.5 Real‐time PCR for pathogen detection

DNA extractions were performed according to the FDA BAM for diarrheagenic *E. coli* (Feng et al., 2020) or *Salmonella* (Andrews et al., 2023). All real‐time PCR analysis was performed on a Quantstudio 3 PCR system (ThermoFisher) using 0.2 mL reaction volumes. Each reaction plate had three no template controls and three positive control wells. For pathogenic *E. coli*, multiplex reactions for *stx1*, *stx2*, and *eae* associated with both STEC and enteropathogenic *E. coli* were carried out using primers adapted from Fratamico et al. (2011), listed in Table S1. Reactions were prepared according to the master mix manufacturer's instructions (Thermo Fisher Scientific, 2021). For *Salmonella*, the *invA* gene was targeted using primers from the BAM (Table S1) using SYBR green Master Mix (ThermoFisher) prepared according to manufacturer instructions (Thermo Fisher Scientific, 2022).

### 2.6 Model inputs

Model inputs are summarized in Table 1. MPN *E. coli* 100 mL^−1^ data at the lower limit of detection (<1 MPN *E. coli* 100 mL^−1^ water) were replaced with 0.5 MPN, and samples at the upper limit of detection (>2419.6 MPN *E. coli* 100 mL^−1^) were replaced with 4839.2, twice the upper limit of detection prior to log10 transformation. Sum of precipitation 1, 3, and 7 days prior to sampling were selected to evaluate impact of precipitation timing.

**TABLE 1:** Variables included in model creation.

Variable	Units	Description	Source
MPN *E. coli* 100 mL^‐1^	log10 MPN	log transformed level of *E. coli* present in water sample	Enumerated at lab using Colilert Quanti‐tray 2000 (IDEXX)
Temperature	(°C)	Water temperature during collection	Collected in situ using YSI Exo 2 sond (YSI)
pH		pH of water where sample was collected	
Turbidity	NTU		
Phycocyanin	RFU		
Chlorophyll‐a	RFU		
Fluorescent dissolved organic matter (fDOM)	RFU		
Dissolved oxygen (DO)	mg L^−1^	Determined using optical sensor (YSI)	
Specific conductance (SPC)	uS cm^−1^		
Nitrogen as nitrate	mg L^−1^	Determined using optical sensor (NitraLED, YSI)	
One‐day precipitation		Sum of precipitation 1 day prior to sampling (exclusive of sampling date)	USDA ARS weather stations or UGA weather network
Three‐day precipitation		Sum of precipitation 3 days prior to sampling	
Seven‐day precipitation		Sum of precipitation 7 days prior to sampling date	
GHI		Sum of global horizontal irradiance (GHI) for 2 × 2 km area including farm address up to noon of sampling day	National Solar Radiation Database (Sengupta et al., 2018)
Pond		Pond where sample was collected	
Month		Month when sample was collected	
Pond location		Combination of pond and location, for example, pond 2 location 14 = 214	
Area		Area where sample was collected from surface, subsurface, or perimeter	

### 2.7 Model creation

RF (“ranger”) (Wright et al., 2022), GLM (regression) and MNR (classification) linear models (“glmnet”) (Friedman et al., 2023), SVM (“kernlab”) (Karatzoglou et al., 2004; Karatzoglou et al., 2024), and KNN (“kknn”) (Schliep & Hechenbichler, 2016) models were built using package “Tidymodels” version 1.2.0 (Kuhn & Wickham, 2020). Package “Future” was used for parallel processing to decrease computation time (Bengtsson, 2020). Regression models were created for the MPN *E. coli* data, while classification models were used for pathogenic *E*. coli and *Salmonella* presence/absence data using the listed algorithms. MPN *E. coli* models were created for each pond as well as for the overall dataset.

Due to the limited data available for the pathogen datasets, models were only created for the overall dataset. All datasets were split 80/20 into training and testing dataset using package “rsample” (Frick et al., 2024) prior to models being built and tuned (tuning is the adjustment of hyperparameters for best performance on the dataset [Feurer & Hutter, 2019]) using the training data, with the testing only used for evaluation to avoid data leakage (Lones, 2021). All data were stratified according to the outcome prior to splitting; in the case of MPN *E. coli*, data were split into quartiles and sampled evenly across those quartiles. All numeric predictors were first power transformed using the Yeo Johnson transformation (Yeo & Johnson, 2000), then normalized to meet assumptions of the SVM and KNN models. Categorical variables, such as factors (location, area) were assigned dummy variables to allow them to be used in SVM and KNN modeling. All zero‐variance variables were removed from analysis. Model preprocessing was done using the “recipes” package (Kuhn et al., 2024).

To address spatial autocorrelation (Meyer et al., 2018), leave‐location‐out cross validation (*v* = 10, repeats = 5) from package “spatialsample” version 0.6.0 (Mahoney et al., 2023) was used for resampling the MPN *E*. coli data. Model hyperparameters were tuned using a 10‐point Latin hypercube search (McKay et al., 1979) to optimize root mean square error (RMSE). The best performing hyperparameters were finalized on each model, which was then fit to the training data then evaluated against the test data.

Because of the limited size and strong imbalance of the pathogenic *E. coli* dataset, traditional cross validation (*v* = 5) instead of leave‐location‐out cross validation was used. Leave‐location‐out (*v* = 5) was used for the less imbalanced *Salmonella* dataset, but no strata was set due to the unequal distribution of *Salmonella* presence. Missing values were imputed using bagged trees with the default parameters (Kuhn & Johnson, 2013). Synthetic minority oversampling (SMOTE), which oversamples rarer class and supplements with synthetic data and undersamples the majority class was used for the pathogen training datasets (Chawla et al., 2002), has been shown to be effective in addressing class imbalance in pathogen presence in water samples (Weller et al., 2021b). SMOTE was applied using the “themis” package (Hvitfeldt, 2025) version 1.0.3 with the over ration and number of neighbors tuned during the hyperparameter search. Model hyperparameters were tuned with a 10‐point Latin hypercube search to optimize receiver operating characteristic (ROC) area under the curve (AUC), then precision recall (PR) AUC.

### 2.8 Model evaluation

RMSE, *R* ^2^, and mean absolute error (MAE) were calculated for each of the mpnEC (regression) models. Since RMSE and MAE are in the same unit as the variable it is measuring, comparison between datasets requires the context of the scale/distribution of what is being measured (in this study MPN *E. coli* 100 mL^−1^) or normalization (normalized root mean square error, normalized mean average error), such as dividing by the range and multiplying by 100 (Stocker et al., 2022). Table S2 gives the descriptive statistics of each dataset, including the minimum and maximum.

ROC AUC, PR AUC, Brier score (Brier, 1950), and miss rate were calculated for each of the pathogen (classification) models. ROC is a measure of the true positive rate to the false positive rate (Fawcett, 2006), and in highly unbalanced datasets, may overestimate a model's performance (He & Garcia, 2009) due to false positives making little impact when compared against a vast number of negatives. PR is a measure of precision (ratio of true positives to true positives and false positives) and recall (ratio of true positives to total positives), both of which consider the number of true positives as well as false positives and false negatives. The AUC for these two metrics indicates their performance. Brier score is the mean square probability error between a prediction (Kruppa et al., 2014): a lower score is better. Miss rate is the ratio of false negatives to false negatives and true positives. The best performing tuned model of each type was fit to the training data and evaluated on the testing data to determine the final performance metrics.

Variable importance was assessed by permutation, as implemented in the package “DALEX” (Biecek, 2018) and “DALEXtra” (Maksymiuk et al., 2020). Variable importance for regression models was based on the RMSE gained when a predictor was permuted. Importance variables were selected based on a cutoff of 0.15 from the full model, determined by the describe.feature.importance.explainer function from package “ingredients” (Biecek & Baniecki, 2023).

## 3 RESULTS

### 3.1 MPN *E. coli* models

#### 3.1.1 All pond dataset

Tuning results for the models based on the complete dataset (*n* = 1266) are displayed in Figure S1. RFs best fit the *E. coli* levels for all observations, followed by SVM, GLM, and KNN (Table 2). Variable importance for RF models was highest for SPC (RMSE gain after permutation = 0.85), precipitation 3 days prior to sampling (0.61), and pH (0.51). For SVM models, variable importance was greatest for 3‐day precipitation (0.78), month (0.72), and pH (0.71). GLM models were dependent most on month (0.87), 3‐day precipitation (0.86), and pond (0.80), while KNN models only had location (1.1) as an important variable.

**TABLE 2:** Most probable number (MPN) *E. coli* model performance.

		*R* ^2^	NRMSE	NMAE
All	**RF**	**0.782**	**11.2**	**7.9**
	SVM	0.61	15	10.6
	GLM	0.672	16.9	10.6
	KNN	0.307	20	15.9
Pond 1	**RF**	**0.64**	**11**	**7.8**
	SVM	0.39	13.8	12.5
	GLM	0.46	13.8	9.28
	KNN	0.17	16.3	12.5
Pond 2	**RF**	**0.72**	**11.3**	**8.28**
	SVM	0.57	14.8	10.8
	GLM	0.56	15	10.3
	KNN	0.29	17.8	13.3
Pond 3	RF	0.67	14.7	10.4
	SVM	0.65	15.3	10.8
	**GLM**	**0.73**	**13.4**	**10.1**
	KNN	0.68	14.7	11.1

*Note*: Bold indicates best model performance by normalized root mean square error (NRMSE). Models used were random forest (RF), support vector machines (SVM), generalized linear models (GLM), and K‐nearest neighbors (KNN). Fit was evaluated using *R* ^2^, NRMSE, and normalized mean average error (NMAE).

#### 3.1.2 Pond 1

Tuning results for the models of the Pond 1 dataset (*n* = 563) are summarized in Figure S2. At Pond 1, RF again best modeled *E. coli* levels, GLM performed better than SVM, and KNN performed the worst (Table 2). The 3‐day precipitation (0.59) and SPC (0.55) variables were the most important for RF models; SPC (0.85), 3‐day precipitation (0.84), month (0.79), pH (0.73), GHI (0.70), 1‐day precipitation (0.61), sampling area (0.61), phycocyanin level (0.59), and chlorophyll level (0.58), were the important variables for GLM. The 3‐day precipitation (0.68), pH (0.67), SPC (0.64), and month (0.60) variables were most important for SVM, and pH (0.74), month (0.67), and 3‐day precipitation (0.67) were most important for the KNN models.

#### 3.1.3 Pond 2

At Pond 2 (*n* = 505), RF models performed the best, followed by SVM, GLM, and KNN (Table 2). Figure S3 shows model tuning. No variables were found to be significantly important (less than 0.15 RMSE dropout difference from the full model) for the RF models. Month (0.68) and DO (0.65), were most important for the SVM models, while eight variables: 3‐day precipitation (2.27), 1 day precipitation (1.8), month (1.3), 7‐day precipitation (0.86), GHI (0.81), NTU (0.75), pH (0.72), SPC (0.67), and phycocyanin concentration (0.67), DO (0.78), and month (0.77) were most important for KNN models.

#### 3.1.4 Pond 3

Model tuning results for Pond 3 (*n* = 198) are summarized in Figure S4. For the fitted models, Pond 3 was the only dataset where a non‐RF model had the best performance: GLM, followed by RF, KNN, and SVM (Table 2). Eight significant variables were identified for the GLM: month (1.2), pH (0.97), 1‐day precipitation (0.79), temperature (0.75), 7‐day precipitation (0.72), GHI (0.61), 3‐day precipitation (0.61), and DO (0.54), and no significant predictors for RF. The most important variable for KNN models was month (0.74), and for SVM, month (0.77), pH (0.71), and temperature (0.55).

### 3.2 Pathogenic *E. coli* models

#### 3.2.1 All pond models

Modeling tuning results for the pathogenic *E. coli* models for the dataset consisting of observations of all ponds (*n* = 717), of which 20 samples were positive for at least one pathogenic gene are summarized in Figure S5. The models’ performance during tuning was inferior to that of the finalized models’ performance with the test data. When tuned to maximize ROC AUC (Figure 2) fit to the training set and evaluating the test set, RF performed best, followed by SVM, multinomial regression, and KNN performed the worst (Table 3). No important variables were found for RF and SVM models. Eleven of the 20 variables for the KNN models were found to be important; the top five were GHI (0.69 ROC AUC loss), pH (0.69), 1‐day precipitation (0.68), phycocyanin content (0.67), and chlorophyll (0.67). For the multinomial regression model, temperature (0.77) and 3‐day precipitation (0.76) were the most important variables.

**FIGURE 2:**
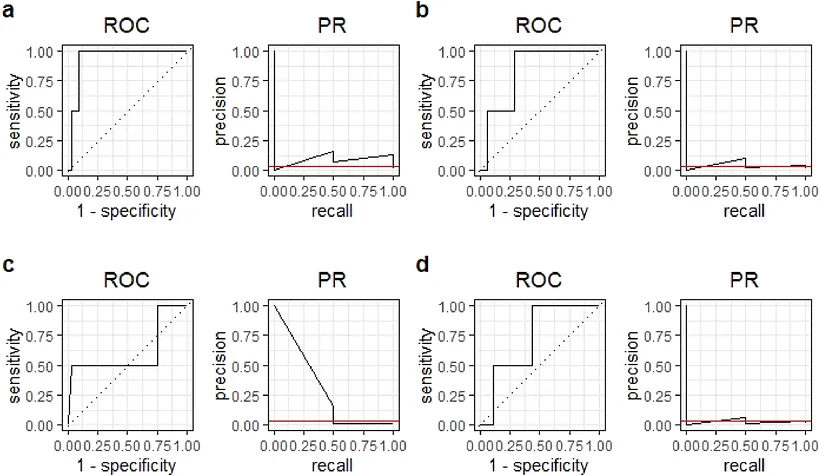
Performance of the pathogenic *E. coli* models, (a) random forest, (b) support vector machines, (c) K‐nearest neighbors, and (d) multinomial regression, tuned to maximize receiver operator characteristic (ROC), showing both ROC and precision recall (PR) curves. Red line on PR curve indicates the level of precision achievable by a random estimator.

**TABLE 3:** Pathogenic *E. coli* model performance for models: random forest (RF), support vector machines (SVM), multinomial regression (MNR), and K‐nearest neighbors (KNN), tuned to maximize the receiver operator characteristic (ROC) area under the curve (AUC) and precision recall (PR) AUC.

	Model	ROC AUC	PR AUC	Brier score	Miss rate
ROC	**RF**	**0.91**	**0.093**	**0.04**	**1**
	SVM	0.82	0.043	0.07	0.5
	MNR	0.73	0.026	0.12	0.5
	KNN	0.61	0.31	0.07	0.5
PR	RF	0.94	0.09	0.04	1
	**SVM**	**0.5**	**0.51**	**0.25**	**0.5**
	**MNR**	**0.5**	**0.51**	**0.25**	**0.5**
	KNN	0.61	0.3	0.07	0.5

*Note*: Bold indicates best model performance by normalized root mean square error (NRMSE).

Of the pathogenic *E. coli* models tuned to maximize PR AUC (Figure 3), SVM performed the best, tied with multinomial regression. KNN did the third best followed by RF (Table 3). Loss of PR AUC after permutation did not identify any important variables.

**FIGURE 3:**
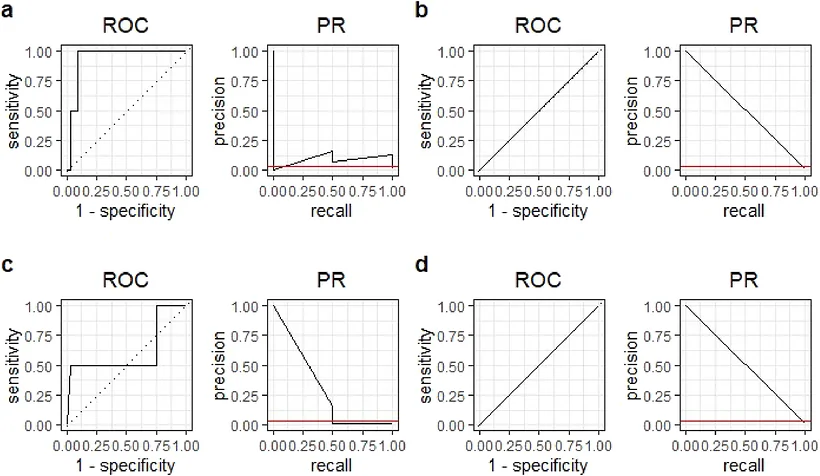
Performance of the pathogenic *E. coli* models, (a) random forest, (b) support vector machines, (c) K nearest neighbors, and (d) multinomial regression, tuned to maximize area under the precision recall (PR) curve, showing both receiver operator characteristic (ROC) and PR curves. Red line on PR curve indicates the level of precision achievable by a random estimator.

### 3.3 *Salmonella* models

#### 3.3.1 All *Salmonella* data

The model tuning results for the dataset with both ponds’ data (*n* = 284), of which 23 were *Salmonella‐*positive is summarized in Figure S6. The best performing model optimizing the ROC AUC (Figure 4) was the multinomial regression with the highest ROC AUC and better Brier score than the SVM and RF models that had higher PR AUC (Table 4). No important variables for multinomial regression, SVM, or RF were determined by permutation. For the KNN model, eight variables were found to be important; the top five were location (0.69 ROC AUC loss), GHI (0.64), 1‐day precipitation (0.63), 7‐day precipitation (0.63), and sampling area (0.62).

**FIGURE 4:**
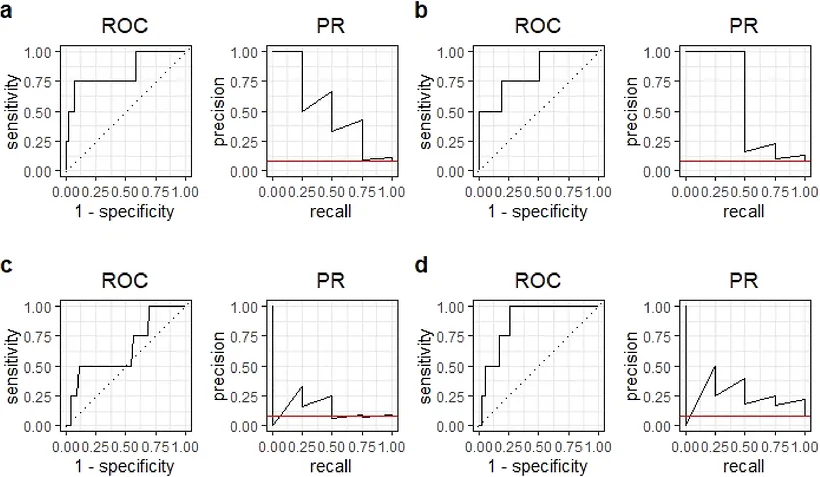
Performance of *Salmonella* models, (a) random forest, (b) support vector machines, (c) K nearest neighbors, and (d) multinomial regression, tuned to maximize receiver operator characteristic (ROC) showing both ROC and precision recall (PR) curves. Red line on PR curve indicates performance of a random classifier.

**TABLE 4:** *Salmonella* model performance for models: random forest (RF), support vector machines (SVM), multinomial regression (MNR), and K‐nearest neighbors (KNN), tuned to maximize the receiver operator characteristic (ROC) area under the curve (AUC) and precision recall (PR) AUC.

	Model	ROC AUC	PR AUC	Brier score	Miss rate
ROC	RF	0.83	0.52	0.06	0.5
	SVM	0.83	0.58	0.08	0.5
	**MNR**	**0.86**	**0.43**	**0.11**	**0.5**
	KNN	0.63	0.09	0.18	0.5
PR	RF	0.84	0.52	0.06	0.5
	SVM	0.79	0.57	0.21	1
	**MNR**	**0.81**	**0.58**	**0.11**	**0.5**
	KNN	0.65	0.25	0.13	0.75

*Note*: Bold indicates best model performance by normalized root mean square error (NRMSE).

When tuned to maximize the PR AUC (Figure 5), multinomial regression performed the best, with highest PR AUC, and lower miss rate than SVM models (Table 4). No significant predictors were identified for multinomial regression, SVM, or RF. For KNN, temperature (0.12 PR AUC loss after permutation) was the only significant predictor.

**FIGURE 5:**
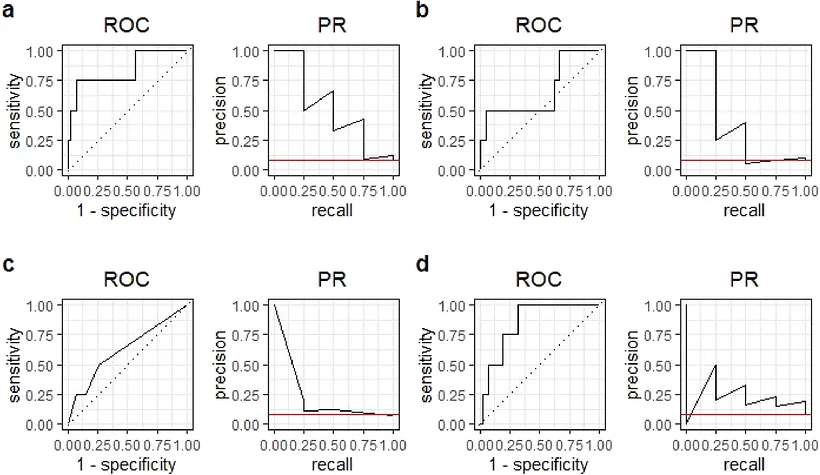
Performance of *Salmonella* models, (a) random forest, (b) support vector machines, (c) K nearest neighbors, and (d) multinomial regression, tuned to maximize precision recall (PR) showing both receiver operator characteristic (ROC) and PR curves. Red line on PR curve indicates performance of a random classifier.

## 4 DISCUSSION

### 4.1 Variable importance

Important predictors for the MPN *E. coli* levels were not consistent between ponds. While SPC and 3‐day precipitation were most important RF models for the overall dataset and Pond 1, none were identified for Pond 2, and 3‐day precipitation, but not SPC, was one of the eight predictors important to the GLM model that performed best on the Pond 3. None of the relatively well‐performing pathogen models identified important variables. KNN analysis for pathogenic *E. coli* identified numerous factors, and multinomial regression identified temperature and 3‐day precipitation, but both models performed poorly (Figure 2). The 3‐day precipitation variable was shared with the MPN *E. coli* models and may reflect a similar runoff effect. *Salmonella* KNN models had important predictors identified by permutation, but were poor performers, limiting the utility of interpreting their feature importance. Location was the only feature that was important for both, which may indicate that there is a location‐specific factor not reflected in the other model inputs that explains the occurrence of *Salmonella* in these ponds, or spatial autocorrelation was not addressed well enough during model creation.

Rainfall was identified as one of the three most important variables in a study in New York predicting *E. coli* levels in water using machine learning (Weller, Love, et al., 2020), and SPC was identified as important more often than any other in a study using machine learning to predict *E. coli* levels in Maryland (Stocker et al., 2022). It also correlated with *E. coli* levels in a study in central Florida (McEgan et al., 2013). Previous work in the same region as this study found a 0.61 log MPN *E. coli* 100 mL^−1^ difference in paired pond samples before and after rain events (Harris et al., 2018), with even higher levels found in runoff feeding the ponds (1.9–2.1 log MPN *E. coli* 100 mL^−1^), which suggests that runoff may be a significant source of *E. coli* in ponds like those in the present study. Coefficients of the GLMs provide context for how factors influence the outcome. The 3‐day precipitation had the largest coefficient, 0.42, while 1‐day precipitation was a negative influence on MPN *E. coli* (−0.10), suggesting that rainfall may dilute the *E. coli* in the water (Kraay et al., 2020; Levy et al., 2009) while also introducing additional *E. coli* through runoff (X. Liu et al., 2025).

Conductivity increases may indicate a disturbance that introduces more solids to the water (EPA, 2025), as with rainfall brining microbial and nutrient load through runoff, and in the Chesapeake Bay watershed, deviations from background conductivity in water were found in areas with agriculture (Fanelli et al., 2024). *E. coli* was shown to have a logarithmic subsidy‐stress response to a modeled river water salinity system (DeVilbiss et al., 2021), but most of the evaluated concentrations were beyond SPC levels seen in this study.

In central Florida, MPN *E. coli* and solar radiation correlated with MPN *Salmonella* in surface water (McEgan et al., 2013), and GHI was identified as one of the significant predictors for KNN. In the Georgia Coastal Plain, increased water temperature associated with *Salmonella* presence (Antaki et al., 2016). Work in New York State found radiation, air temperature, DO, *E. coli*, and pH among other predictors increased the odds of *Salmonella* being isolated in a sample (Weller, Belias, et al., 2020), while in the southeast, humidity, nitrate, calcium, and solar radiation were among the most important variables for detection of STEC genes in water samples (Chevez et al., 2024). While increasing temperature is associated with decreased *E. coli* survival time in agricultural water (Blaustein et al., 2013), in the GLM for the complete dataset, December has a negative coefficient (−0.05) and June (0.35) had an opposite effect, indicating that factors beyond temperature drove differences by month.

### 4.2 Model performance

RF models performed best on all but one of the MPN *E. coli* datasets, but only one of the pathogen datasets. This could be a result of the larger dataset played to RF's advantages, particularly avoiding overfitting on the test data, while multinomial regression's penalization helped quiet noise in the smaller pathogen datasets (Friedman et al., 2010; Kuhn & Johnson, 2013). RF models’ performance did not respond much to tuning (Probst et al., 2019) in contrast to SVM, where performance depended greatly on the tuning of the kernel (Koch et al., 2012).

ROC AUC values for pathogenic *E. coli* models were comparable to other studies of STEC in water, which have been reported as 0.72 (Weller, Bellias, et al., 2020), 0.850 (Ceuppens et al., 2015), 0.91–0.92 (Belias et al., 2021), and 0.921 (Chevez et al., 2024). The precision of the best performing *Salmonella* models was comparable to a study using artificial neural networks, KNN, and SVM to model the occurrence of *Salmonella* in surface waters in central Florida (Polat et al., 2020). The difference in the models tuned to maximize ROC AUC and PR AUC highlights that a model catered to increasing one metric does not necessarily lead to an increase in the other (Davis & Goadrich, 2006). For imbalanced data, such as the 2.7% positive rate in the pathogenic *E. coli* data, ROC becomes less informative, and PR becomes the appropriate measure of performance (He & Garcia, 2009; Saito & Rehmsmeier, 2015; Sofaer et al., 2019). Models may have desirable ROC curves (a perfect ROC curve is a 90° curve capturing an AUC of 1), but a PR curve close to random (Boyd et al., 2012). Focusing only on the AUC of the PR curve also has its disadvantages. While the multinomial regression may have performed better than the RF by PR AUC, examining the PR curve (Figure 5) shows the model has poor initial recall indicating more missed true positives, undesirable in a pathogen prediction setting.

The two best performing pathogenic *E. coli* models tuned to maximize the PR AUC were both “fence sitting” models—effectively guessing randomly (Goldstein‐Greenwood, 2021), as reflected by the ROC AUC of 0.5 and Brier score of 0.25 (Figure 3). All the pathogen models in this study had poor miss rates: the best being 0.50, and often failing to properly categorize any of the true positive samples as positives. This is unacceptable in the context of agricultural water management, as it may lead to inaction when a pathogen is potentially present.

The poor performance of the models for pathogenic *E. coli* may be a result of the class imbalance, a lack of strong predictors, or methodology: individual isolates were analyzed instead of a volume of water, though this study's detection rate aligned with STEC occurrence in isolates recovered from 10 L of water (Haymaker et al., 2019). The occurrence of *Salmonella* in this study (23/284) was slightly lower than similar work in the region (34/285) (Antaki et al., 2016), which may reflect differences in methodology (MMS vs. membrane filtration) or the low levels of *Salmonella* in these systems (max concentration Antaki et al. (2016) observed was 1.80 MPN *Salmonella* 100 mL^−1^ in subsurface pond water).

### 4.3 Utility and regulatory context

Drivers of *E. coli* level in this region may differ by site, and trends identified in larger datasets may not be applicable to individual ponds. This work highlights one of the benefits of longitudinal data collection for agricultural water: it allows a grower to generate trends and see when their agricultural water source has a deviation from trend that would require investigation and correction. Measurements like SPC and precipitation identified as relevant to MPN *E. coli* levels in this study have the advantage of providing real‐time information, while microbiology may require days of turn‐around and marginal cost versus the fixed cost of a conductivity meter, or freely available weather data.

The models generated in this work were not effective predictors of foodborne pathogen presence in agricultural water. While *E. coli* is an imperfect predictor of pathogen presence (Havelaar et al., 2017; Luo et al., 2015; Pachepsky et al., 2016), trends in *E. coli* levels provide a dataset that can demonstrate control over an agricultural water system, for example, showing limited change in *E. coli* levels after a change in adjacent land use.

### 4.4 Study limitations

These models were based on three ponds and are geographically limited in scope. The pathogen presence datasets were strongly imbalanced, and techniques like SMOTE, which may offer performance benefits over other techniques like oversampling may cause an overestimation of predictive abilities, particularly of weak classifiers (Elor & Averbuch‐Elo, 2022; Kuhn & Johnson, 2013). The strong class imbalance leads to poorer estimation of model performance by some metrics. SMOTE was selected to reduce imbalance during model training, because so few positives were available that undersampling alone would have left a small training set, so the trade‐off of model performance overestimation and overfitting was made. Including a robust set of evaluation metrics, such as PR and miss rate in addition to ROC and Brier score helps temper overestimations of model performance. Permutation as a measure of feature importance may be affected by correlation between predictors or nonsensical observations created through permutation (e.g., a water temperature of 13°C in July) (Molnar, 2024). While spatiotemporal autocorrelation was addressed with leave‐location‐out cross validation for the MPN *E. coli* models, the limited datasets for pathogens did not allow for this correction.

## 5 CONCLUSION

This work did not consistently identify any easily measurable or observable factors for robust prediction of pathogen occurrence in irrigation ponds in the Georgia Coastal Plain. While some conditions, such as the cumulative precipitation prior to sampling, were identified that increased both the expected level of *E. coli* and were important to a model's ability to predict a the occurrence of a pathogen, these trends were often observed on models that did not perform well. The identification of spatial and temporal variables as important in many of the models highlights the utility of longitudinal data collection, even in a small system like irrigation ponds. This work also demonstrates the difficulty in identifying measurable factors that indicate that a foodborne pathogen is reasonably likely to occur in a water source.

## AUTHOR CONTRIBUTIONS

**J. Andrew Widmer**: Data curation; formal analysis; investigation; validation; visualization; writing—original draft; writing—review and editing. **Matthew Stocker**: Methodology; resources; software; validation. **Jaclyn E. Smith**: Methodology; resources. **Oliva Pisani**: Investigation; resources. **Alisa Coffin**: Investigation; resources. **Manan Sharma**: Funding acquisition; investigation; methodology; writing—review and editing. **Yakov Pachepsky**: Conceptualization; funding acquisition; investigation; methodology; resources; supervision; visualization; writing—review and editing. **Laurel L. Dunn**: Methodology; project administration; supervision; writing—review and editing.

## CONFLICT OF INTEREST STATEMENT

The authors declare no conflicts of interest.
